# Influence of Binders and Lightweight Aggregates on the Properties of Cementitious Mortars: From Traditional Requirements to Indoor Air Quality Improvement

**DOI:** 10.3390/ma10080978

**Published:** 2017-08-22

**Authors:** Chiara Giosuè, Mattia Pierpaoli, Alessandra Mobili, Maria Letizia Ruello, Francesca Tittarelli

**Affiliations:** 1 Department of Materials, Environmental Sciences and Urban Planning (SIMAU), Università Politecnica delle Marche, 60131 Ancona, Italy; c.giosue@univpm.it (C.G.); m.pierpaoli@pm.univpm.it (M.P.); a.mobili@univpm.it (A.M.); m.l.ruello@univpm.it (M.L.R.); 2 Institute of Atmospheric Sciences and Climate, National Research Council (ISAC-CNR), 40129 Bologna, Italy

**Keywords:** mortar, cement, lightweight aggregates, Indoor Air Quality (IAQ), depollution, moisture buffering value

## Abstract

Innovative and multifunctional mortars for renders and panels were manufactured using white photocatalytic and non-photocatalytic cement as binder. Unconventional aggregates, based on lightweight materials with high specific surface and adsorbent properties, were adopted in order to investigate the possible ability to passively improve indoor air quality. The reference mortar was manufactured with traditional calcareous sand. Results show that even if the mechanical properties of mortars with unconventional aggregates generally decrease, they remain acceptable for application as render. The innovative mortars were able to passively improve indoor air quality in terms of transpirability (70% higher), moisture buffering ability (65% higher) and depolluting capacity (up to 75% higher) compared to traditional ones under the current test conditions.

## 1. Introduction

The impact of the building sector in terms of global energy consumption is about 40% [1]. Therefore currently in this sector, laws and regulations are stricter in terms of energy efficiency: buildings are more air-tied and the indoor air quality (IAQ) worsens inevitably [2]. On the other hand, in recent decades, changes in lifestyle have led people to spend 70–90% of their time indoors [3] where they often come into contact with an unhealthy environment. Occupants are potentially exposed to airborne pollutants such as Volatile Organic Compounds (VOCs), anhydrides (NO_x_, SO_x_), and ozone (O_3_) [4] which can have both short- and long-term effects on human health, leading to the well-known Sick Building Syndrome (SBS).

Also, non-adequate levels of Relative Humidity (RH) have negative effects on the comfort and well-being of the occupants. The optimal RH level is about 50% [5]: RH levels below 25% cause discomfort and drying of the mucous membranes and skin [6], high RH levels cause discomfort and favour biological growth [7,8,9]. 

Conventionally, the broad strategies to reduce indoor pollutants or to modulate RH are: source control, dilution by active engineered control systems (Heating, Ventilation and Air-Conditioning—HVAC) and stand-alone air purifiers [10]. Source control is very difficult since the occupants and furnishings are sources themselves. Active systems require energy to function affecting energy consumption in buildings. Building materials such as mortars, plasters and finishes, thanks to their high surface in indoor applications, can interact with the indoor microclimate as passive systems, increasing IAQ without energy demand [11,12,13,14]. 

Depolluting properties of building materials can be due to their adsorption capacity and/or to their photocatalytic activity (PCA). Cementitious materials enriched with TiO_2_ in bulk [15] or as coating [16,17] show self-cleaning [18] and depolluting properties thanks to the photocatalytic effect of TiO_2_ under UV irradiation. Horgnies et al. (2012) described how the addition of a low quantity of activated carbon in a photocatalytic cementitious matrix can enhance the decomposition of NO_x_, improving the adsorptive phase of the process [19]. For indoor applications, Vieira et al. (2014) added commercial mortars with TiO_2_, superabsorbent polymers and Phase Changing Materials (PCMs) to improve the depolluting properties, Moisture Buffering Capacity (MBC) and thermal insulation [13]. The authors studied that the application of a TiO_2_-based paint on different support materials enhances the degradation of NO and selected VOCs [20]. Tittarelli et al. (2015) and Giosuè et al. (2016) investigated the ability of mortars to passively improve indoor comfort and health, in terms of indoor pollutants adsorption and MBC, by using unconventional adsorbent aggregates and biomass waste materials [21,22].

In the current research, the aim was to investigate the effect of using both unconventional adsorbent aggregates and a cementitious photocatalytic binder on the ability of the mortars to passively improve indoor comfort and health. The transport of the pollutant over the activated catalyst surface is the first step of heterogeneous photocatalysis. By using an adsorbent material as aggregate, the pollutant concentration in the proximity of the catalyst is increased, perhaps enhancing the photocatalytic efficiency. For this purpose, sixteen types of mortars were manufactured with three different adsorbent aggregates, using both a white photocatalytic and non-photocatalytic cement, as reference. A commercial calcareous sand was used as reference aggregate. Mortars were compared in terms of mechanical strength, capillary water absorption, shrinkage, water vapour permeability, MBC and depolluting properties. Since water promotes degradation of building materials, with a consequent increase of maintenance costs, hydrophobic agents are often adopted in mortar formulations to improve durability, due to their ability to make concrete less susceptible to water saturation [23]: the possible effect of a hydrophobic admixture on mortar properties was also investigated. 

## 2. Materials

### 2.1. Binders

White cement CEM II A/LL 42.5 R (C) was used as a reference binder. White photocatalytic cement (PC), thanks to its patented photocatalytic admixture [24,25], of the same class strength was used as a photocatalytic binder.

### 2.2. Aggregates

**Figure 1:**
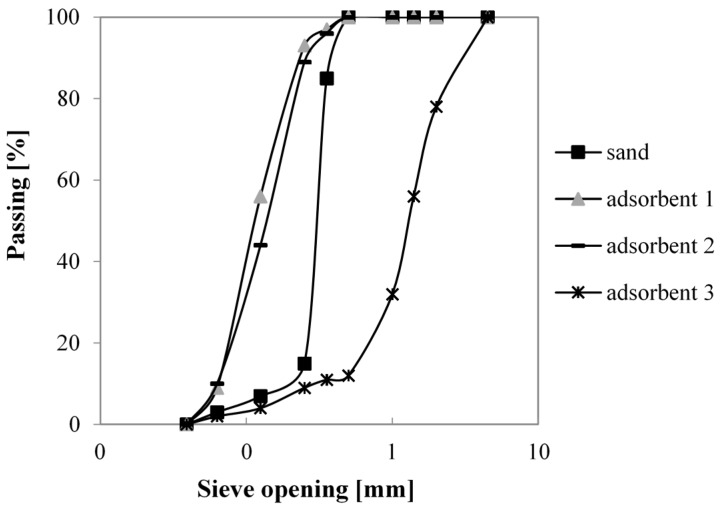
Grain size distribution curves of aggregates.

The adopted unconventional aggregates, indicated as A1, A2, A3, are lightweight materials generally used as adsorbent for heavy metals, dyes, oil, and molecular sieves in gas separation processes. A1 and A2 are polar adsorbents, while A3 is a non-polar adsorbent. Table 1 compares their physical properties, obtained by data sheets and laboratory tests, with those of a natural commercial sand used as reference. Figure 1 provides the particle size distribution curves of aggregates obtained by sieving.

### 2.3. Admixtures

A hydrophobic admixture (h), based on a 45% water solution of butyl-ethoxy-silane was added during the cast at the dosage of 1.2% by binder weight in some mixtures in order to have a hydrophobic bulk mortar. 

### 2.4. Mix Design

Table 2 shows the mix proportions of mortars. The dosages of aggregates are reported in saturated surface dried (ssd) condition. Reference mortars were manufactured with cement (white and photocatalytic) and commercial sand (C-S and PC-S), with (h) and without hydrophobic admixture. Mortars have a water to binder ratio (w/b) of 0.5 by weight and an aggregate to binder ratio (a/b) of 3.5 by volume. The tested mortars were manufactured by replacing 100% sand volume with different unconventional aggregates. Before casting, aggregates were water pre-soaked till constant mass was achieved. 

## 3. Methods

### 3.1. Workability

The mortar workability was measured by flow table, according to UNI EN 1015-3:2007.

### 3.2. Mechanical Properties

Compressive strength (*R_c_*) tests were carried out according to the Italian standard UNI EN 1015-11:2007 on at least three prisms (40 × 40 × 160 mm) cured at T = 20 ± 2 °C and RH = 95 ± 3% for 7 days and then at T = 20 ± 2 °C and RH = 65 ± 3% for the following 21 days. A ‘Galdabini’ hydraulic press with a precision of 1% was used. The average results are reported. 

After 28 days of curing, the density (*ρ*, in kg/m^3^) of hardened mortars was calculated and the specific strength, one of the most common parameters used for comparing materials [26], was evaluated as *R_sc_* = *R_c_*/*ρ* (Pa/(kg/m^3^)). 

From the results of compressive strength, Elastic modulus *E_c_* was evaluated according to the empirical relation suggested by the ACI Committee (ACI 318-95) [27] *E_c_* = 4.73(*f_c_*)^1/2^ (GPa), where *f_c_* is the cylindrical compressive strength of mortars evaluated with the relation *f_c_* = 0.83·*R_c_*, proposed by [28].

### 3.3. Microstructure: Morphology and Pore Size Distribution

In order to correlate macroscopic properties with microstructure, the morphology of specimens was investigated by Scanning Electron Microscopy (SEM); a ZEISS 1530 SEM (Carl Zeiss, Oberkochen, Germany) equipped with a Schottky emitter, with two different secondary electrons (SE) detectors (the in-lens and the Everhart-Thornley) and operating at 10 keV was used. Two small mortar fragments of about 1 cm^3^ were collected from each specimen and covered with a thin layer of graphite to make them conductive before one observation. The most representative SEM images of the mortars were then reported.

Moreover, in cementitious mortars the porosity can be divided in: (a) gel pores—nano-pores inside the hydration products, with pore diameter of about 0.5–10 nm; (b) capillary pores—micro-pores between the hydration products, with pore diameter between 10 nm and 1 µm, strongly dependent on the hydration degree and the w/b; (c) macro pores—pores due to entrained air with spherical micro-bubbles, with pore diameters higher than 10 µm and (d) porosity into the aggregate [28].

In this case, aggregate porosity strongly influences the properties of mortars; the unconventional aggregates have additional nano porosity compared to conventional sand. The effect of unconventional lightweight aggregates on the pore size distribution of mortars was studied by Mercury Intrusion Porosimetry (MIP) with Mercury Porosimeter PASCAL 240 (Thermo Fisher Scientific, Waltham, MA, USA). For each mortar typology, three small mortar fragments of about 1 cm^3^ were tested after 28 days of curing and the average results are reported.

### 3.4. Drying Shrinkage

Mortars applied in an environment at RH < 95% are subjected to drying shrinkage. The free drying shrinkage of different mortars was measured according to UNI EN 12617-4:2003. 

Three prismatic specimens (40 × 40 × 160 mm) were cured at T = 20 ± 2 °C and RH = 95 ± 3% for the first 24 h. Immediately after demoulding, the initial length of the sample was recorded and then exposed at T = 20 ± 2 °C and RH = 50 ± 5%. Sample length and weight were recorded for at least 40 days by using a comparator with a measuring accuracy of 0.001 mm and a scale with an accuracy of 0.01 g, respectively. The average results are reported.

### 3.5. Capillary Water Absorption

Capillary water transports aggressive agents and deteriorates mortars [29] therefore the study of capillary water absorption is of primary importance to test the durability of a construction material. 

The capillary water absorption of mortars is measured according to UNI EN 1015-18:2004. Three test specimens were obtained by prisms (160 × 40 × 40 mm) broken into two halves, and cured as described in Section 3.2. Specimens were placed in a box, semi-immersed in water at a depth of 5 to 10 mm, by keeping constant the level of water during the test. The box was kept closed to prevent water evaporation. Specimens were removed from the box after 10 min, the surface was quickly dried with a moist fabric to remove the excess of water and then specimens were weighed and immediately placed back into the box. The procedure was repeated after 90 min. The capillary water absorption coefficient (*C*) of mortar is the average between the specimens and it is calculated according to the standard.

### 3.6. Water Vapour Permeability 

Mortars and renders should have a good water vapour permeability to facilitate the drying process of the masonry assemblage, as well as the disposal of water vapour produced inside buildings [30].

Water vapour permeability measurements are carried out according to the UNI EN 1015-19:2007 and data processed according to UNI EN ISO 12572:2007. Three cylindrical mortar specimens (d = 12.5 cm; h = 3.0 cm), cured as described in Section 3.2, were placed on the top of a sample-holder with a saturated solution of potassium nitrate (KNO_3_) inside (RH = 93 ± 3% at T = 20 ± 2 °C). The containers were placed in a climatic chamber at T = 20 ± 2 °C and RH = 50 ± 5% and the test started. On the side surface, the specimens were sealed with a non-breathable film in order to guarantee the unidirectional flow of the water vapour from inside to outside, due to the difference of RHs. The mass of the specimens was monitored day by day until stationary conditions were achieved. The average results of the test are reported in terms of the water vapour diffusion resistance factor, *µ*. *μ* is defined as the ratio between the vapour permeability of stagnant air *δ_a_* (kg/(Pa m s)) and the vapour permeability of the material *δ_p_* (kg/(Pa m s)) at the same temperature and pressure [31].

### 3.7. Moisture Buffering Capacity

Indoor environments are subjected to quick changes in RH and building materials should act as a buffer for moisture. The Moisture Buffering Capacity (MBC) is the capacity of a material to absorb and release moisture from/to the environment where it is placed [32]. 

In this paper, the influence of unconventional aggregates on MBC of mortars is assessed by a simplified version of the NORDTEST method [33] where specimens are cyclically exposed to different RHs for fixed periods. Three cylindrical specimens (d = 10.0 cm, h = 3.0 cm) were manufactured and cured as described in Section 3.2. Before testing, specimens were pre-conditioned in a climate chamber at T = 20 ± 2 °C and RH = 50 ± 3% until constant weight was achieved. In order to enhance the aggregate contribution to the moisture buffering properties of mortars, the specimens were superficially abraded and cleaned with compressed air before testing. 

Specimens were exposed to cyclic step-changes that alternate high levels (75% for 8 h) and low levels (33% for 16 h) of RH. The test simulates daily variations. The exposure to the two different RHs was carried out by putting the specimens inside two climate boxes, respectively containing a saturated solution of magnesium chloride (MgCl_2_, RH = 33%) and sodium chloride (NaCl, RH = 75%). The boxes were kept inside a climatic chamber to maintain the temperature constant at T = 20 ± 2 °C during the test. The duration of the entire cycle was 24 h. The amount of water vapour absorbed or released by the specimens during each step was determined by measuring the weight of the specimens before changing boxes [21].

The practical Moisture Buffering Value (*MBV*) (g/(m^2^% RH)) is calculated as the amount of moisture changed by the material per surface unit and RH gradient, as indicated in [34].

### 3.8. Depolluting Properties

Depolluting properties of different mortars were evaluated by two different tests. The first one consists of a continuous flow test and the second one is a batch test, respectively to investigate the photocatalytic activity towards the nitrogen oxide (NO) removal and the VOC. Also in this case, in order to enhance the aggregate contribution on the depolluting properties, the specimens were superficially abraded and cleaned before testing. 

#### 3.8.1. Continuous Flow Test

In the continuous flow test, nitrogen oxide (NO) was chosen as a model of air pollutants. NO is commonly found in indoor air. The photochemical smog reactions result from the interaction of various pollutants such as NO_x_ and/or VOCs in the presence of sunlight. 

For this reason, anthropogenic NO_x_ emission constitutes a wider problem in indoor conditions than in outdoor ones and its removal is considered beneficial to prevent the formation of secondary pollutants [25]. 

The continuous flow test was performed according to the procedure provided by the Italian standard UNI 11247:2010. The specimen (cylinder with d = 9 cm and cured as described in Section 3.2) was placed inside a borosilicate glass chamber of 3.58 L on a tripod in order to irradiate the surface with UVA radiation provided by an UVA metal-halogen quartz lamp with mercury vapour, peak at 360 nm and adsorbed power of 400 W. The distance between the surface of the sample and the lamp guarantees a specimen radiance of 20 W/m^2^ (photoradiometer HD2102.2, with a probe centered in the field of UVA, Delta OHM, Padova, Italy). The inlet gas was a mixture of synthetic air and NO. The chamber was linked to a nitrogen oxides analyser. The NO inlet concentration was 500 ppb (flux of 1.5 L·min^−1^) and the abatement coefficient *A_C_*, in terms of the percentage of abated NO, was evaluated according to the standard.

#### 3.8.2. In-Batch Test 

In the adsorption test, methyl-ethyl-ketone (MEK) is chosen as model of air pollutants (VOCs) for its environmental stability. This compound is an irritant for human eyes and nose and harmful health effects occur at high concentrations, Threshold Limit Value (TLV) = 200 ppm = 590 mg/m^3^. The MEK vapour pressure is 95.1 mmHg at T = 25 °C. It has an odour threshold of 5.4 ppm, corresponding to 16 mg/m^3^ [35]. 

The concentration of MEK in a 16.65 L sealed borosilicate glass box containing the specimen was monitored over time [21]. Inside the test box a fan guarantees continuous air recirculation. The specimens are cylindrical (d = 3.4 cm, h = 4 cm) and cured as described in Section 3.2. Air samples inside the box were collected by a micro-syringe and analysed with a gas chromatograph (Flame Ionization Detector, injector split 1:15, carrier flow 2 mL/min, capillary column, 25 m × 0.32 mm, 0.52 μm cross linked methyl siloxane, isotherm condition 40 °C). The MEK injected into the test box was initially 50 µL corresponding to 2402 mg/m^3^ (approximately 4 times TLV). Three measurements were repeated and the average results of the test are reported. The data starts to be consistent 20 min after the initial injection when all MEK has been vaporised. Then, the results were plotted as a percentage of detected concentration (*C_i_*) divided by to the initial concentration (*C*_0_). Tests were carried out under two different conditions: under dark conditions and under 10 W/m^2^ UVA radiation on the surface of the specimens.

## 4. Results and Discussion

### 4.1. Workability

Slump values are shown in Table 2. The use of unconventional aggregates in ssd condition instead of conventional sand permits mortars to be obtained with the same stiff workability, according to UNI EN 1015-6:2007, since the slump flow is always lower than 140 mm, with no evidence of segregation. Also, the hydrophobic admixture does not affect workability. 

### 4.2. Mechanical Properties

Results are shown in Table 3. The use of different binders does not imply differences in compressive strength results. As already reported in the literature, a slight decrease in compressive strength is generally observed with the addition of a hydrophobic admixture [36,37].

The main differences were measured with different aggregates. Mortars prepared with A2 aggregate show the lowest value of mechanical strength, about 80% lower than sand mortars. In the case of A3 mortars, the mechanical strength is about 45% lower than sand mortars due to the lowest density (Table 1). The best results are recorded by A1 mortars where values are even slightly higher (5%) than those of the reference mortar, in spite of the significant (20%) lower density compared to the reference. Being directly evaluated from the mechanical strength, the *E_c_* values show the same trend.

Thanks to the total replacement of calcareous sand volume with unconventional aggregate A2, lightweight mortars can be obtained. The value of the density is ≤1300 kg/m^3^, which is the limit of classifying mortars as lightweight according to UNI EN 998-1:2010. A lightweight material is highly appreciated in non-structural applications, since it gives benefits in terms of the lower weight of the structure, higher sound absorption, and lower costs of buildings [38,39].

A3 mortars have the same *R_sc_* value as the reference mortar, A2 mortars have four times less specific resistance than sand mortars but, thanks to the increase in lightness, A1 mortars have about 35% higher value of *R_sc_* than the reference mortars. 

Also in this case, there are no relevant differences in using different binders or adding the hydrophobic admixture. 

### 4.3. Microstructure: Morphology and Pore Size Distribution 

**Figure 2:**
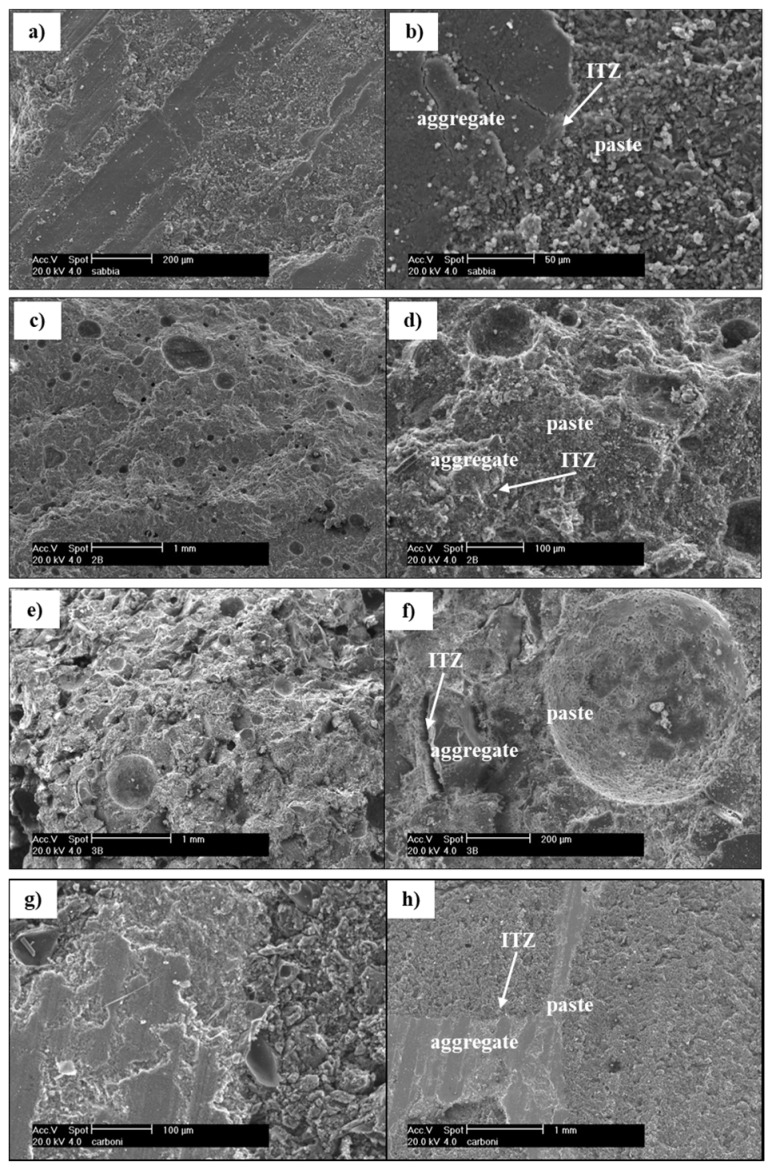
Scanning electron microscopy (SEM) images of mortars: (**a**,**b**) C-S; (**c**,**d**) C-A1; (**e**,**f**) C-A2; (**g**,**h**) C-A3.

Figure 2 reports the morphological observations obtained by SEM analysis of different mortars.

The microstructure of the reference mortar (Figure 2a,b) appears homogeneous and dense. This, together with the high mechanical strength of calcareous sand and the good adhesion observed between sand and cementitious paste, explains the high mechanical performance of the reference mortar. In the case of A1 mortar (Figure 2c,d), the microstructure appears quite homogeneous and dense, even if some entrapped air bubbles are evident. However, the Interfacial Transition Zone (ITZ) between cement paste and A1 aggregate is detectable only with difficulty, meaning there is a good adhesion between aggregate and binder paste. This is probably due to the possible (at least superficially) pozzolanic reactivity of this unconventional aggregate with Ca(OH)_2_ of the cement paste, as already reported by other authors [39]. This justifies the high mechanical behaviour of this mortar, even compared to the conventional sand mortar, in spite of the higher lightness. When A2 aggregate is used (Figure 2e,f), the microstructure of mortar appears very heterogeneous and porous. Some entrapped air bubbles are evident and also the ITZ between A2 aggregate and cement paste is easily detectable meaning a poor adherence between the two components. This is most probably due to the smooth surface of this aggregate. These facts explain why A2 mortar shows the worst mechanical performances. 

With A3 aggregate (Figure 2g,h), mortars have a quite porous but homogeneous microstructure. Moreover, the ITZ between A3 aggregate and cement paste is good. This explains why its mechanical strength is significantly higher compared to that of A2 mortars, even if still lower than that of the reference mortars.

**Figure 3:**
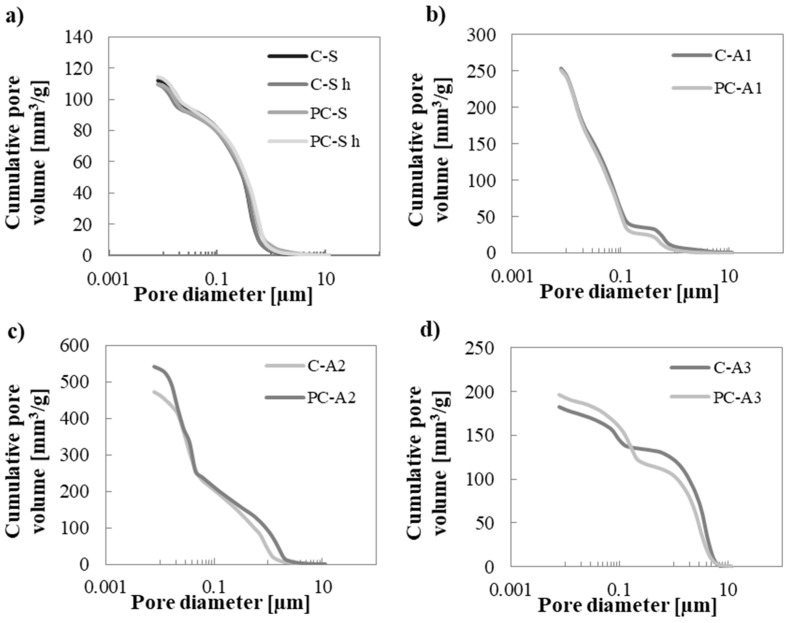
Cumulative pore volume distribution of mortars without hydrophobic admixture: (**a**) C-S, C-S h, PC-S, PC-S h; (**b**) C-A1, PC-A1; (**c**) C-A2, PC-A2; (**d**) C-A3, PC-A3.

Since the introduction of the hydrophobic admixture does not significantly change the mechanical strength of the materials, Table 4 and Figure 3 compare the results of MIP analysis obtained by all sand-based mortars and by unconventional aggregate mortars, but only without hydrophobic admixture.

The higher the specific surface of the unconventional adsorbent aggregate, the higher is the value of the mortar specific surface. This was verified only in A3 mortars; A3 aggregate has a high quantity of pores smaller than 0.01 µm [40] and this range of porosity is too small to be detected by MIP.

Sand mortars have, as expected, the lowest percentage of voids (about 20%). A3 mortars have about 25% more total porosity than the reference mortar. Despite A1 mortars showing the best compressive strength, the total pore volume of these mortars is about 36%, 75% higher than the sand mortars. As already explained, the optimum transition zone observed by SEM analysis (Figure 2c,d), and the possible pozzolanic activity explains their excellent mechanical behaviour. A2 mortars have the highest value of total porosity (*Vp* ~ 50%), more than two times higher than that measured in sand mortars. Together with the bad ITZ between A2 aggregate and cement paste observed by SEM images (Figure 2e,f), their higher porosity explains their low compressive strength.

Figure 3 shows the MIP results in terms of cumulative pore volume distribution. The curves generally have a poly-modal distribution with two main bends: the first is around pore diameters of 0.430 µm, the second around pore diameters of 0.013 µm. The bend at the lower diameter is related to the paste because it is detected in all specimens; the second bend is characteristic of each aggregate. In case of A1 mortars, porosity distribution becomes tri-modal. Bends are at 0.43 µm, 0.08 µm and 0.01 µm, respectively. For A2 and A3, mortar bends are at 0.93 µm and 0.21 µm and 3.27 µm and 0.1 µm, respectively.

The addition of the hydrophobic admixture does not generate differences in terms of pore size distribution. If the photocatalytic binder is used, pores are slightly shifted towards smaller diameters. Also in a previous study [41,42], the introduction of 5% in weight of TiO_2_ in mortars shifted pores dimension to smaller values. This trend is not detected in A2 mortars, perhaps due to the high total porosity (Table 4) of the mortar itself, mostly introduced by the poor ITZ (Figure 2f). 

### 4.4. Drying Shrinkage

**Figure 4:**
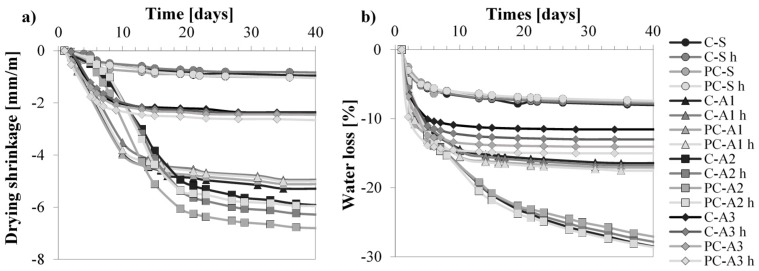
Mortars exposed at RH = 50% and T = 20 °C: (**a**) drying shrinkage; (**b**) water loss.

Sand mortars have the lowest value of drying shrinkage and water loss (Figure 4); after seven days the specimens do not show significant length variation.

**Figure 5:**
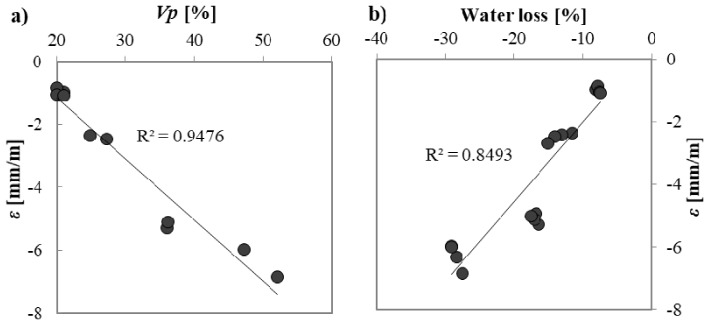
Correlation between (**a**) shrinkage and total porosity; (**b**) shrinkage and water loss of mortars.

In general, when sand is replaced by unconventional aggregates, the drying shrinkage is enhanced. The use of A3 implies 2.5 times higher values of shrinkage. When A1 and A2 are used, mortars shrink five and seven times more than the reference, respectively. This behaviour is related to the higher water loss of unconventional mortars due to their higher total porosity compared to the reference. In fact, Figure 5 reports the correlations found between shrinkage (*ε*) at 40 days and total pore volume and between ε at 40 days and water loss: the higher the total porosity and higher the shrinkage; the higher the water loss, the higher the shrinkage.

Drying shrinkage in mortars depends not only on the amount of total porosity but also on the dimension of pores [43]: the lower the pore radius, the higher the induced stress due to the evaporation of a certain amount of water. In this case, A1 and A2 mortars have the highest quantity of pores at small diameters (Figure 3). Despite A3 mortars having a lower quantity of pores with smaller diameters than the reference mortars, they have higher shrinkage due to the higher amount of total open porosity *Vp*, which permits higher water evaporation.

**Figure 6:**
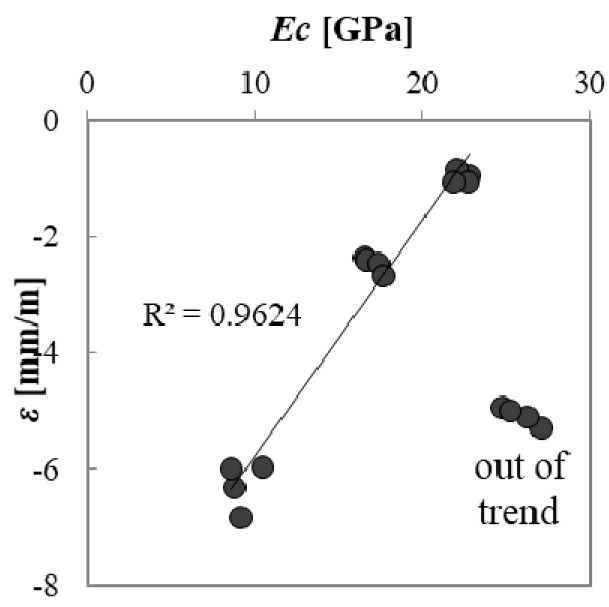
Correlation between drying shrinkage at 40 days and modulus of elasticity.

Shrinkage is also related to the *E_c_* of mortars: the higher the *E_c_*, the lower the value of shrinkage for a certain stress. Sand mortars have the highest *E_c_* value and, therefore, the lowest shrinkage value. A quite good linear correlation is found between shrinkage and calculated *E_c_* except for A1 mortars (out of trend dots in Figure 6) where the high *E_c_* is due to the high mechanical resistance, in spite of the high porosity (Table 3 and Table 4). 

There are no relevant changes in drying shrinkage due to different binders or the hydrophobic admixture.

### 4.5. Capillary Water Absorption

**Figure 7:**
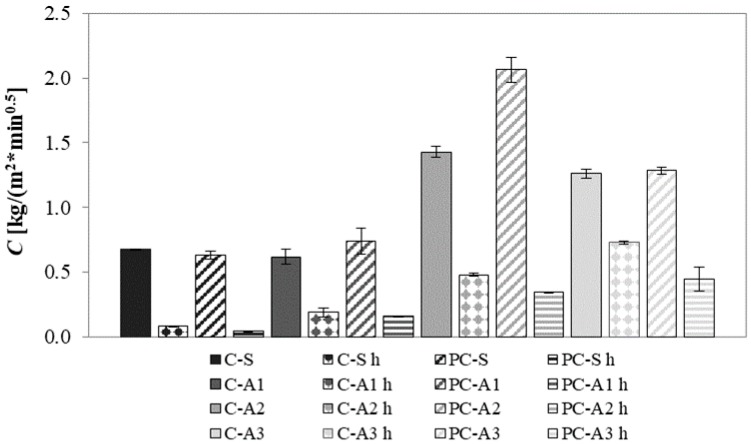
Capillary water absorption coefficient of mortars.

Generally, as expected, the hydrophobic admixture is the main factor controlling the water uptake (Figure 7). The open porosity of mortars is the second most influential parameter: the higher the open porosity, the higher the water amount that can fill the pores. Therefore, the use of unconventional aggregates increases the water uptake of mortars due to their higher porosity, even if by a different amount (Table 4). 

The photocatalytic binder slightly increases water absorption: usually, hydrophilicity is given to mortars by TiO_2_ addition [44,45]. 

In sand mortars, the use of a hydrophobic admixture reduces more than eight times the water uptake. In mortars with unconventional aggregates, the effectiveness of the hydrophobic admixture is lower. This can be explained by the great specific surface of these mortars (Section 4.3) that cannot be well covered by the same quantity of silane.

In the case of A1 mortars, the water uptake is only slightly higher than that of sand mortars. The hydrophobic admixture reduces about four times the water uptake. A2 mortars absorb the highest quantity of water, three times higher than sand mortars, due to their higher porosity (Table 4). Moreover, A2 is a polar adsorbent and the great affinity of this type of adsorbent with water is well-known [46]. However, the use of a hydrophobic admixture decreases about five times the *C* value. A3 mortars have a *C* value two times higher than sand mortars. A3 is a non-polar adsorbent but is not considered a hydrophobic material, since in saturation condition the water absorption is comparable to that of other hydrophilic materials [46]. Moreover, in spite of the A3 mortar total porosity (26%) being lower than that of A1 mortars (36%), capillary water absorption is higher due to the increased presence of larger pores. The initial (linear) phase of capillary water absorption corresponds to the filling of the larger capillary pores, while the second (non-linear) phase corresponds to the filling of the smaller pores [47,48]. Since a short time test (90 min) is used, large pores are more influential on capillary water absorption than small ones. In this case, the use of hydrophobic admixture decreases the *C* value by about two times. 

### 4.6. Water Vapour Permeability

**Figure 8:**
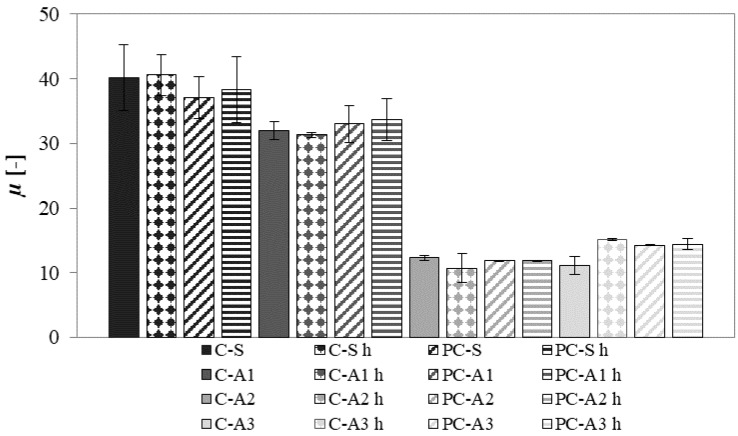
Water vapour resistance factor *µ* of mortars.

Water vapour permeability results, expressed in terms of *µ* factor, are shown in Figure 8. Lower values of *µ* factor indicate higher values of permeability. 

For all mortars, the use of photocatalytic or white cement and the hydrophobic admixture does not affect the results. The use of different aggregates is the main influencing factor.

Sand mortars have the highest value of *µ* meaning the lowest water vapour permeability. A1 mortars show *µ* value 20% lower than the reference mortar. A2 and A3 mortars have a very low *µ* value, approximately 70% and 60% lower than that of sand mortars, respectively. 

The permeability to water vapour depends on many factors, the principle in this case is the open porosity. Sand mortars are the less porous and less permeable to water vapour. However, according to Poiseuille’s law, permeability depends not only on open porosity but also on pore size (permeability should be proportional to the product of porosity and square diameter of the main mode pore), connectivity, and tortuosity of the microstructure [49]. In particular, the greater the threshold pore diameter, the higher the permeability of the material [50]. A2 mortars are the most porous and A3 mortars have the largest pore diameters. These facts explain why A2 and A3 mortars are the most permeable, with *µ* comparable to those obtained in hemp-lime mortars (*µ* ~10) [51]. 

### 4.7. Moisture Buffering Capacity

**Figure 9:**
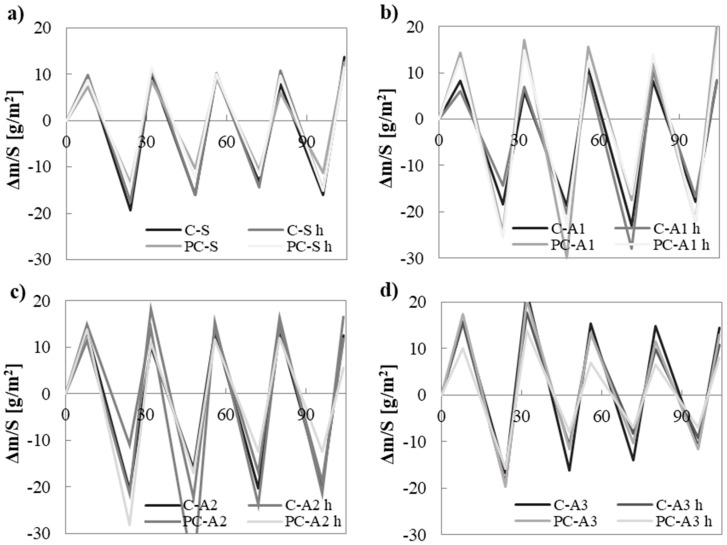
Change in water vapour content of mortars normalised on the surface of the specimens: (**a**) sand based mortars; (**b**) A1 based mortars; (**c**) A2 based mortars; (**d**) A3 based mortars.

Figure 9 shows the change in water vapour content (Δm) normalised on the exposed surface of the specimens at different RH values. 

The use of different binders or hydrophobic admixture does not affect this behaviour. On the contrary, the use of different aggregates is again the main influencing factor.

Sand mortars have the smallest exchange of water vapour. A1 and A2 mortars adsorb and desorb water vapour about two times more than sand mortars. A3 mortars have a slightly higher (about 20%) capacity to adsorb/desorb water vapour than sand mortars. 

**Figure 10:**
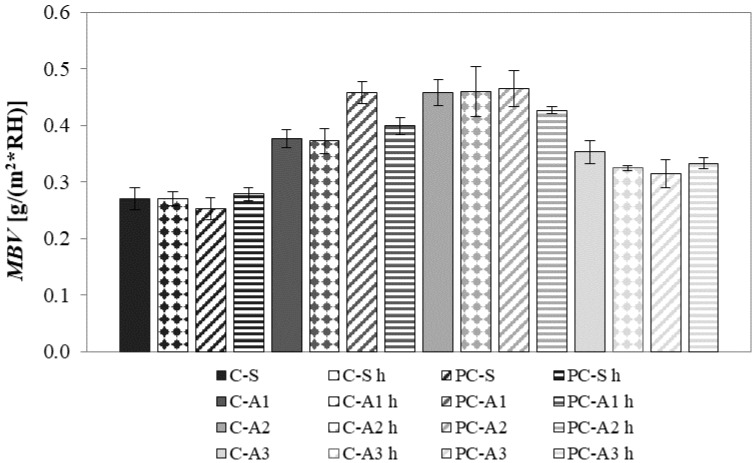
Moisture buffering value (*MBV*) of mortars.

The higher the specific surface of mortars, the higher is the ability of the material to uptake/release water vapour [52]. A2 mortars have the highest percentage of porosity and specific surface (Section 4.3) and show the best ability to change water both at low and high RHs [46] in terms of *MBV* (Figure 10). Moreover, the great affinity of A2 polar adsorbent with water is well known. A2 mortars are followed by A1 and then A3 mortars. A1 mortars are less porous than A2 ones. Also, A3 mortars are less porous than A2. Moreover, A3 is a non-polar adsorbent, with lower affinity to polar molecules of water than A1 and A2 [46,53]. 

### 4.8. Depolluting Properties

#### 4.8.1. Continuous Flow Test

**Figure 11:**
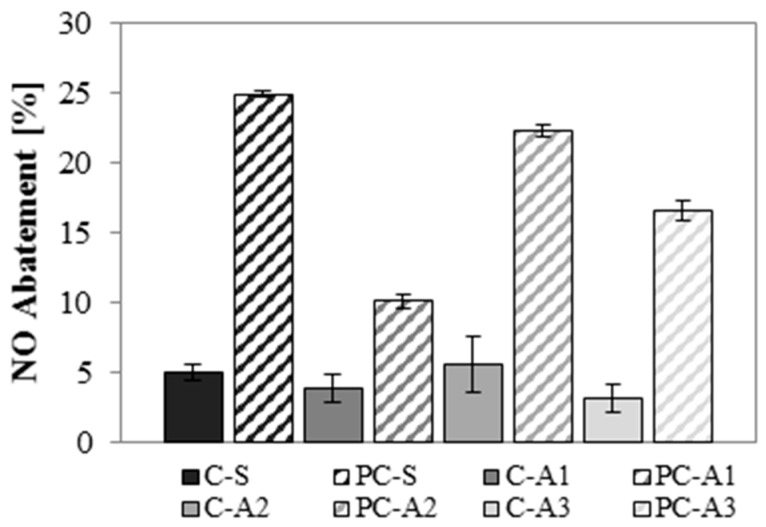
Photocatalytic efficiency under UVA radiation of mortars.

Results of the photocatalytic efficiency of mortars, tested in terms of NO abatement under UVA radiation, are shown in Figure 11.

NO decomposes only in the presence of photocatalytic binder. The slight abatement of NO detected when white cement is used, is ascribed to the presence of a small amount of photocatalytic agent [54]. In this test, the adsorption process can be excluded: since the test starts when the stationary flux condition is reached, the substrate has enough exposure time to be saturated. With the same test, and adopting similar initial conditions of flux and NO concentration (550 ppb instead of 500 ppb), quite similar abatements (around 20%) have been reported [25]. 

Sand mortars give the best NO abatement. However, A2 mortars show NO abatement comparable to that of sand mortars (~22%). A3 mortars reduce NO abatement by about 30% compared to the sand mortars. A3 aggregate has a black colour: dark colours could reduce the reflectance of radiation, increasing its absorption [55]. The worst interaction between the photocatalytic binder and unconventional aggregate is detected in A1 mortars, where the efficiency is about 40% of that measured in reference mortars. A1 is an adsorbent material with a high specific surface and high micro-porosity but it enhances the hydration products thanks to its pozzolanic activity (Section 4.3). New hydration products can increase compressive strength (Section 4.2) but can cover the active TiO_2_ sites [56], decreasing the photocatalytic efficiency of mortars.

#### 4.8.2. In Batch Test

**Figure 12:**
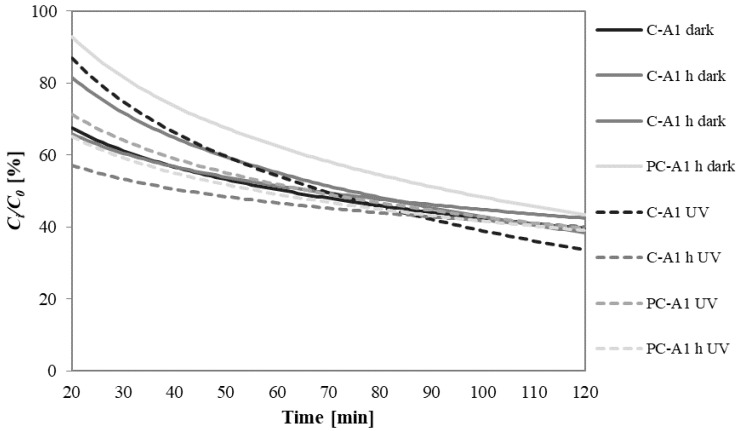
Methyl-ethyl-ketone (MEK) residual concentration inside the box as a function of time under dark conditions and under UVA radiation: A1 mortar.

As example, Figure 12 reports the residual percentage of MEK inside the box with A1 mortars as a function of time, under dark conditions and under UVA radiation. The trend line starts at 20 min because this time is necessary for the complete vaporization of MEK (Section 3.8.2).

**Figure 13:**
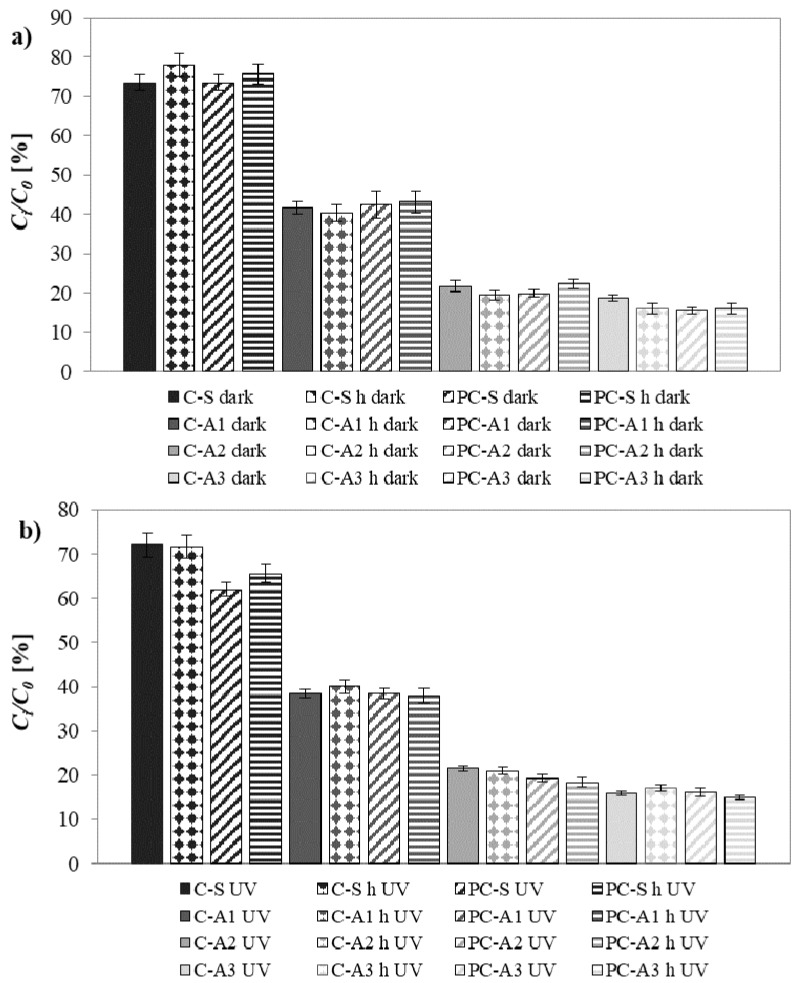
MEK residual concentration inside the box after 120 min testing: (**a**) under dark condition; (**b**) under UVA radiation.

To better compare the results, Figure 13 shows the MEK residual percentage inside the box with different mortars, performed under dark conditions and under UVA radiation, after 120 min testing.

The obtained values are comparable to those already reported in the literature with the same method [21,22]. Generally, the use of a hydrophobic admixture does not influence the depolluting properties of mortars. The main influencing factor is again the different typology of aggregates.

Sand mortars left 70% of MEK inside the box. In the presence of the photocatalytic binder, under UVA radiation, there is an increase in removal efficiency of about 15%. A1 mortars, thanks to their high porosity, have almost a double depolluting capacity, with MEK residual of about 40%. A2 and A3 mortars have depolluting capacity even four times higher than traditional sand mortars: the residual MEK concentration is only 20% after 120 min of test. These high efficiencies can be explained by the high specific surface of A2 mortars and with the great affinity of non-polar A3 aggregate with organic molecules as MEK [53,57]. 

In the presence of unconventional aggregates, no significant increase in MEK removal is observed in the presence of photocatalytic binder under UVA radiation. This means that the adsorption process of the aggregate predominates on the photocatalytic action of the binder under UVA radiation.

## 5. Conclusions

New mortars for indoor renders or panels manufactured with three different types of lightweight unconventional aggregates (A1, A2, A3), in order to enhance their ability to passively improve IAQ, were studied. Unconventional aggregates fully replaced traditional calcareous sand by volume in cementitious mortars where white non-photocatalytic and photocatalytic cements were used as binders. To improve durability, the effect of a hydrophobic admixture on the properties of mortars was also discussed.

The obtained results show that the photocatalytic cement influences only the depolluting properties, while the hydrophobic admixture influences only the water absorption properties of the mortars. 

Nevertheless, all the properties of the mortars were strongly influenced by the use of different aggregates. In particular, when conventional sand was replaced by volume with an unconventional aggregate in mortars, under the current tests methodologies:

- compressive strength decreases, except for A1 mortars where it even increases, but remains still acceptable for non-structural applications as renders;
- lightness is increased: A2 mortar can be classified as a lightweight mortar (density ≤1300 kg/m^3^);
- drying shrinkage increases, but it could be easily reduced by a shrinkage reducing admixture and/or an expansive agent;
- capillary water absorption increases but the hydrophobic admixture reduces it up to five times;
- permeability to water vapour increases up to 70% compared to the reference mortar;
- moisture buffering capacity increases up to 65%;
- depolluting capacity, in terms of MEK removal, increases up to 75%.

No increase in MEK removal was observed in the presence of photocatalytic binder under UVA radiation meaning that, with these unconventional aggregates, the adsorption process of the aggregate predominates on the photocatalytic action of the binder.

## Figures and Tables

**Table 1:** Properties of aggregates.

Aggregate	Code	Specific Surface (m^2^/g)	Density ssd * (kg/m^3^)	Water Absorption after 24 h (%)
Sand	S	5	2650	5
Adsorbent 1	A1	600	1600	22
Adsorbent 2	A2	750	1310	86
Adsorbent 3	A3	900	1180	31

* ssd: saturated surface dried condition.

**Table 2:** Mix proportions (kg/m^3^) and workability of mortars.

Mix	Water	White Cement	Photocatalytic Cement	S	A1	A2	A3	Hydrophobic Admixture	Slump (mm)
C-S	256	512	-	1535	-	-	-	-	120
C-S h	253	512	-	1535	-	-	-	6	115
PC-S	256	-	512	1535	-	-	-	-	120
PC-S h	253	-	512	1535	-	-	-	6	115
C-A1	256	512	-	-	927	-	-	-	108
C-A1 h	253	512	-	-	927	-	-	6	107
PC-A1	256	-	512	-	927	-	-	-	106
PC-A1 h	253	-	512	-	927	-	-	6	106
C-A2	256	512	-	-	-	759	-	-	128
C-A2 h	253	512	-	-	-	759	-	6	122
PC-A2	256	-	512	-	-	759	-	-	124
PC-A2 h	253	-	512	-	-	759	-	6	128
C-A3	256	512	-	-	-	-	683	-	110
C-A3 h	253	512	-	-	-	-	683	6	110
PC-A3	256	-	512	-	-	-	683	-	110
PC-A3 h	253	-	512	-	-	-	683	6	111

**Table 3:** Mechanical properties of mortars: compressive strength (*R_c_*), hardened density (*ρ*), compression specific strength (*R_sc_*), and Elastic modulus (*E_c_*) after 28 days of curing.

Mix	*R_c_* (MPa)	*ρ* (kg/m^3^)	*R_sc_* (Pa/(kg/m^3^))	*E_c_* (GPa)
C-S	31.2 ± 1.0	2091 ± 63	14.92	22.76
C-S h	29.2 ± 1.7	2069 ± 84	14.11	22.02
PC-S	31.0 ± 0.5	2141 ± 52	14.48	22.69
PC-S h	28.9 ± 1.2	2092 ± 49	13.81	21.91
C-A1	32.9 ± 2.1	1677 ± 32	19.62	23.37
C-A1 h	29.5 ± 1.8	1621 ± 21	18.20	22.13
PC-A1	30.9 ± 0.5	1670 ± 27	18.50	22.65
PC-A1 h	28.5 ± 1.0	1609 ± 36	17.71	21.76
C-A2	6.6 ± 0.3	1223 ± 13	5.40	10.47
C-A2 h	4.6 ± 0.1	1192 ± 22	3.86	8.74
PC-A2	5.0 ± 0.3	1202 ± 23	4.16	9.11
PC-A2 h	4.4 ± 0.1	1258 ± 12	3.50	8.55
C-A3	16.4 ± 1.0	1350 ± 51	12.15	16.50
C-A3 h	16.7 ± 0.8	1304 ± 25	12.81	16.65
PC-A3	18.1 ± 0.5	1353 ± 31	13.38	17.34
PC-A3 h	18.7 ± 0.6	1370 ± 34	13.65	17.62

**Table 4:** Total percentage of pores (*Vp*) and specific surface of mortars measured by mercury intrusion porosimetry (MIP).

Mix	*Vp* (%)	Specific Surface (m^2^/g)
C-S	21	7.36
C-S h	20	8.27
PC-S	20	7.54
PC-S h	21	8.08
C-A1	36	39.88
PC-A1	36	40.47
C-A2	47	49.00
PC-A2	52	56.11
C-A3	25	6.69
PC-A3	27	7.80
